# A virtual reality-based multimodal framework for adolescent depression screening using machine learning

**DOI:** 10.3389/fpsyt.2025.1655554

**Published:** 2025-09-17

**Authors:** Yizhen Wu, Yuling Qiao, Licheng Wu, Minglin Gao, Tsz Yiu Wong, Jingyun Li, Zhimeng Wang, Xu Zhao, Hui Zhao, Xiwang Fan

**Affiliations:** ^1^ Clinical Research Center for Mental Disorders, Shanghai Pudong New Area Mental Health Center, School of Medicine, Tongji University, Shanghai, China; ^2^ Department of Primary Education, The New Bund School Attached to No.2 High School of East China Normal University, Shanghai, China; ^3^ Faculty of Psychology, Beijing Normal University, Beijing, China

**Keywords:** virtual reality, multimodal sensing, adolescent depression, EEG, eye tracking, heart rate variability, machine learning, support vector machine

## Abstract

**Background:**

Major depressive disorder (MDD) in adolescents poses an increasing global health concern, yet current screening practices rely heavily on subjective reports. Virtual reality (VR), integrated with multimodal physiological sensing (EEG+ET+HRV), offers a promising pathway for more objective diagnostics.

**Methods:**

In this case-control study, 51 adolescents diagnosed with first-episode MDD and 64 healthy controls participated in a 10-minute VR-based emotional task. Electroencephalography (EEG), eye-tracking (ET), and heart rate variability (HRV) data were collected in real-time. Key physiological differences were identified via statistical analysis, and a support vector machine (SVM) model was trained to classify MDD status based on selected features.

**Results:**

Adolescents with MDD showed significantly higher EEG theta/beta ratios, reduced saccade counts, longer fixation durations, and elevated HRV LF/HF ratios (all p <.05). The theta/beta and LF/HF ratios were both significantly associated with depression severity. The SVM model achieved 81.7% classification accuracy with an AUC of 0.921.

**Conclusions:**

The proposed VR-based multimodal system identified robust physiological biomarkers associated with adolescent MDD and demonstrated strong diagnostic performance. These findings support the utility of immersive, sensor-integrated platforms in early mental health screening and intervention. Future work may explore integrating the proposed multimodal system into wearable or mobile platforms for scalable, real-world mental health screening.

## Graphical Abstract

Graphical abstract of the experiment.

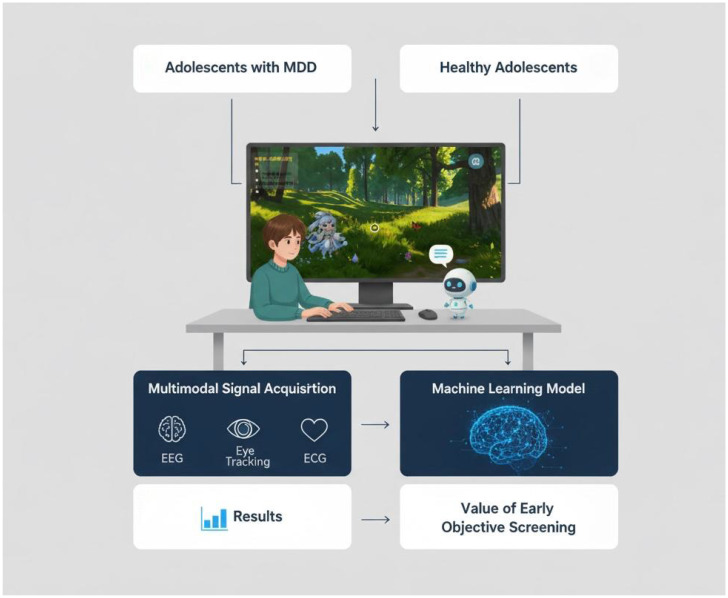

## 1 Introduction

Depression is a widespread mental health disorder characterized by persistent low mood, cognitive impairments, and increased risk of suicide (1). It has become a major global public health concern (2), affecting over 300 million individuals worldwide. Experts project that depression will become the leading contributor to the global disease burden by 2030 (3, 4). Alarmingly, its prevalence among adolescents has more than doubled in recent years (5), in which one in four adolescents globally exhibit depressive symptoms—a rate that surpasses that observed in adult populations (5). In particular, the *2020 Report on National Mental Health Development* in China underscores the severity of the issue, reporting a 24.6% detection rate of adolescent depression, including 7.4% classified as severe cases (6). These statistics reflect the rapidly escalating incidence of adolescent depression and its association with detrimental outcomes.

Despite the growing urgency, current diagnostic approaches for adolescent depression in China remain inadequate. They are predominantly based on symptom checklists and clinical interviews, which lack biological grounding and are susceptible to subjectivity. According to empirical research (7), over 50% of depression cases are either misdiagnosed or overlooked, significantly compromising treatment effectiveness. This underscores the urgent need for objective, biologically grounded diagnostic tools that are independent of self-report or observer bias. Such tools could serve as “emotional lie detectors,” enabling early and accurate detection of depressive states.

As advocated by an empirical study (8), the integration of artificial intelligence (AI) tools into mental health servces will significantly exhibit favorable and effective outcomes. Recent advances in AI and wearable technology offer promising avenues for more objective mental health assessments and interventions. For instance, techniques such as electroencephalography (EEG), heart rate variability (HRV), and eye-tracking (ET) have revealed distinctive neurophysiological and behavioral patterns in individuals with mental disorders compared to healthy controls (9–11). However, several challenges remain. For instance, machine learning models based on frontal EEG alpha-band asymmetry have achieved classification accuracies as high as 88.6% for depressive symptoms (12–14), yet translating these findings into clinically meaningful interpretations—such as understanding frontal lateralization—remains a hurdle (15). Similarly, while HRV-derived metrics reflect autonomic nervous system dysregulation linked to depression severity, results are often confounded by comorbidities such as cardiovascular conditions (16). Eye-tracking studies suggest that individuals with depression exhibit attentional biases toward negative stimuli; however, diagnostic accuracy is limited by inter-individual variability in smooth pursuit and saccadic eye movements (17–19). These limitations reinforce the need for novel screening paradigms that mitigate subjectivity and enhance early detection precision.

Virtual reality (VR) presents a compelling solution by enabling the integration of multimodal data within standardized, immersive environments. VR allows for the seamless collection of behavioral and physiological metrics—such as body movement, gaze patterns, and biosignals—without disrupting user engagement (20, 21). Unlike traditional media, VR’s interactive nature fosters deeper cognitive, social, and physical involvement, thereby enhancing the reliability of psychological assessments (22). Prior research (23, 24) has shown strong correlations between VR-derived metrics and established diagnostic criteria for disorders including schizophrenia, ADHD, and OCD. However, the application of VR in the context of adolescent depression remains underexplored, with no existing standardized frameworks—highlighting a crucial gap in the field.

In response to this gap, our study introduces a pioneering VR-based machine learning framework for screening adolescent depression. By synchronously capturing EEG, HRV, and ET data during user interaction within a VR environment, we aim to identify robust biomarkers of depressive states. This approach seeks to establish a more objective and scalable diagnostic model for adolescent depression, transcending the limitations of traditional methods. Ultimately, this technology-driven paradigm has the potential to transform early identification and intervention strategies for adolescent mental health.

The contributions can be listed as:

Proposes a novel VR-based multimodal framework for adolescent depression screening, integrating EEG, HRV, and eye-tracking (ET) to overcome subjectivity in traditional diagnostic methods.

Develops a machine learning model leveraging synchronized neurophysiological and behavioral data from VR immersion to identify depression biomarkers with higher objectivity than symptom-based assessments.

Addresses a critical gap in adolescent mental health diagnostics by pioneering standardized VR paradigms tailored to this population, where existing tools are scarce or reliant on self-report.

Enhances early detection accuracy by combining immersive environmental control with real-time biosignal analysis, mitigating confounders like comorbidities or inter-individual variability.

Lays groundwork for scalable, technology-driven interventions by demonstrating the clinical translatability of VR and machine learning in mental health screening.

## 2 Materials and methods

### 2.1 Study design and participants

This case-control study was conducted at Weng'an Middle School. From April 2023 to October 2024, a total of 51 adolescents diagnosed with Major Depressive Disorder (MDD) according to DSM-5 criteria (25) and free from psychiatric medications were recruited as the experimental group. While the control group comprised 64 healthy controls (HCs), who were recruited between November 6 and November 20, 2024. All controls underwent standardized psychological screening to confirm the absence of psychiatric history. Ethical approval was granted by the Shanghai Pudong New District Medical Ethics Committee (Approval No. PDJW-KY-2022-011GZ-02), and written informed consent was obtained from all adolescent participants, along with assent from their guardians as required.

The sample size for this study was determined through a power analysis - G*Power 3.1 (26) based on prior neurophysiological research in adolescent depression. Assuming a medium-to-large effect size (*d* = 0.6–0.8) for group differences in EEG, ET, and HRV metrics (9), a minimum of 50 participants per group was required to achieve 80% power (*α* = 0.05, two-tailed). Our final sample (MDD: n = 51; HC: n = 64) exceeded this threshold, aligning with recommendations for machine learning-based biomarker studies (27) and ensuring robust feature selection via RFECV. This sample size is comparable to validated VR-based psychiatric assessments (28, 29) and sufficient to detect clinically meaningful correlations (e.g., theta/beta ratio vs. CES-D: *r* = 0.575, *p* <.001) after Bonferroni correction.

Due to time and funding constraints, participants were recruited from a specific region of China. However, this does not necessarily limit the generalizability of the research. First, our study focuses on MDD, which exhibits high cross-cultural consistency in diagnosis and core symptoms (25, 30). Since DSM-5 criteria are internationally recognized, our findings may extend to other populations, though cultural variations in symptom expression warrant further investigation. Moreover, many psychological phenomena are not strongly influenced by cultural factors. Several other studies with similar participant backgrounds have demonstrated high external validity in subsequent research (31–34). Thus, our research offers a foundational contribution to this field and can guide future cross-cultural validations.

### 2.2 Materials

This study employed a multimodal assessment framework combining validated clinical instruments with advanced neurophysiological monitoring techniques. Depression severity was assessed using the Chinese version of the Center for Epidemiologic Studies Depression Scale (CES-D) (35). Physiological data—including electroencephalography (EEG), ocular motility, and electrocardiogram (ECG)—were recorded using the BIOPAC MP160 system and a See A8 portable telemetric ophthalmoscope.

The virtual reality (VR) environment was embedded within immersive scenarios designed to enhance ecological validity while preserving experimental rigor. A custom VR environment that was developed using the A-Frame framework (36), served as the foundation for the virtual scenes. The system integrated the Claude API (37) to enable dynamic therapeutic dialogue and was delivered through computers. This configuration created a psychologically interactive VR space that supported the detection of depressive symptoms by combining immersive virtual experiences with the natural language capabilities of large language models (LLMs) (38).

As indicated in **Table 1**, the VR immersive setting consisted of panoramic background representing a magical forest by a lakeside, combined with the animated appearance of an AI agent named “Xuyu.” The agent’s dialogue prompts were derived from a standardized script to ensure consistency across participants and reproducibility of the task. Besides, environmental features such as the panoramic background, ambient effects, and the agent’s visual presentation remained constant across all sessions, ensuring controlled conditions. Regarding the task details, participants engaged in interactive dialogues with the agent, who actively initiated conversations around themes of personal worries, distress, and hopes for the future. The overall experience was structured with a fixed duration of approximately 10 minutes, during which the sequence of interaction included an initial greeting, guided emotional exploration, supportive responses, and optional reflective or hopeful exchanges. By creating a secure and private context, participants could express recent emotional distress and future aspirations, which may serve as valuable diagnostic indicators for depression screening.

**Table 1:** Summary of task parameters.

Category	Parameter	Details
Task Structure	Total Duration	10 minutes
	Phases	1. Introduction (1 min) 2. Immersive Relaxation (5 min) 3. Supportive Interaction (3 min) 4. Conclusion (1 min)
Stimuli	Source	Relaxation scenarios
AI Agent	Name	“Xuyu”
Interaction	Participant Input	Verbal responses (audio-recorded)
Environment	Visual Scene	Panoramic magical forest (static lighting/colors)
	Audio	Ambient forest sounds (55 dB, no sudden changes)
Data Sync	Physiological Alignment	LabStreamingLayer (LSL) timestamps for EEG/ET/ECG
	Event Markers	Stimulus onset, dialogue transitions, response triggers

### 2.3 Procedure

**Figure 1:**
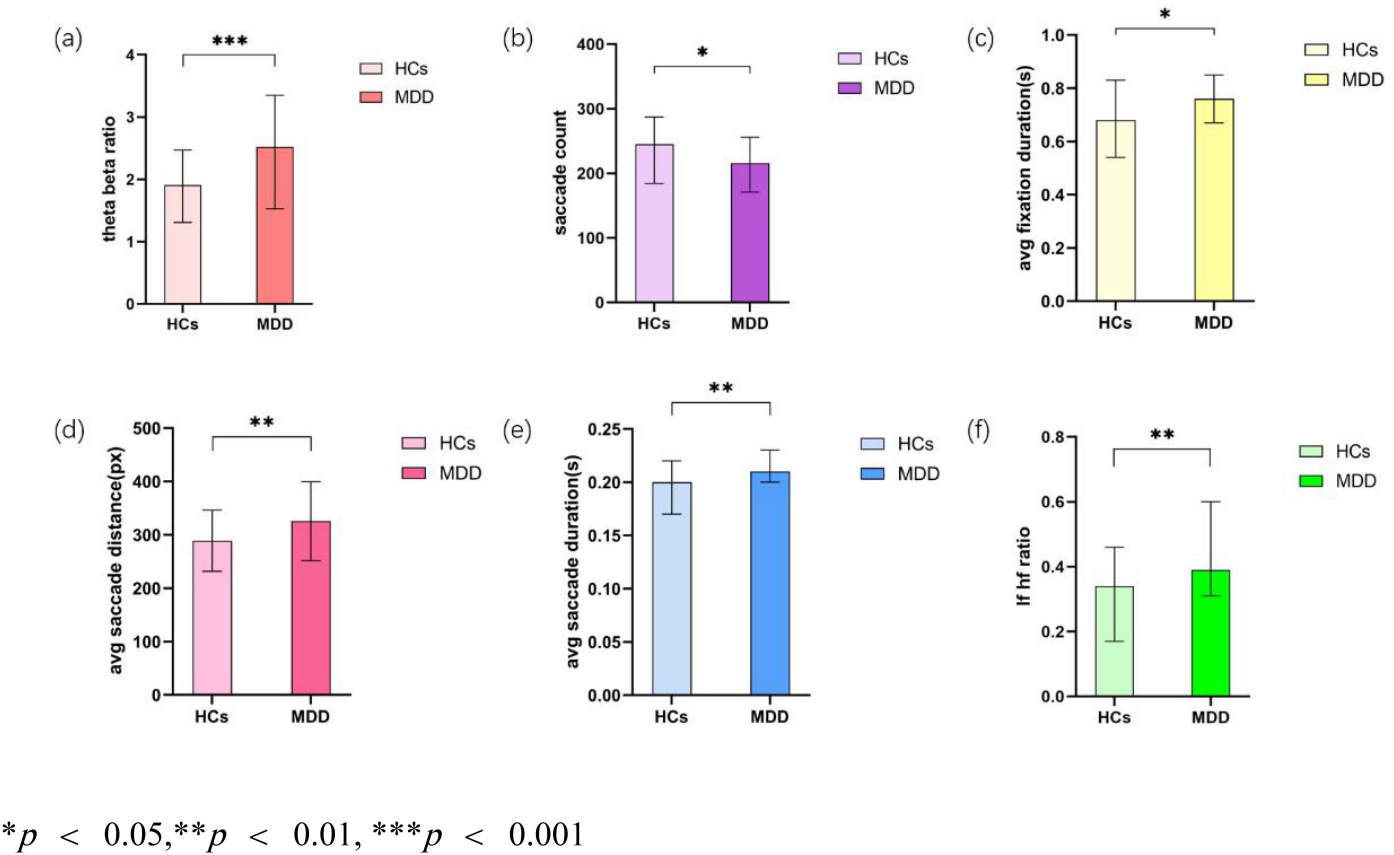
Analysis of key Indicators. **(a)** Theta beta ratio; **(b)** Saccade count; **(c)** Average fixation duration (s); **(d)** Average saccade distance (px); **(e)** Average saccade duration (s); **(f)** LF/HF ratio.

The whole procedure is presented in the graphical abstract below (**Figure 1**). All participants completed the experimental protocol in a sound-attenuated laboratory with standardized ambient lighting conditions. Following initial demographic and clinical assessments, participants were exposed to a 10-minute VR session during which physiological data were continuously recorded.

### 2.4 Data processing and analysis

EEG data were preprocessed through linear interpolation, band-pass filtering between 0.5–100 Hz, and 50 Hz notch filtering to remove powerline noise. Artifacts were removed using discrete wavelet transform (DWT) (39). Eye-tracking data, sampled at 60 Hz, were cleaned by removing outliers, standardizing features, and imputing missing values using either mean or median estimates, as appropriate.

Statistical comparisons between the major depressive disorder (MDD) and healthy control (HC) groups were performed using independent-sample t-tests for normally distributed variables, and Mann–Whitney U tests (40) for non-parametric data. A two-tailed significance threshold was set at p < 0.05. Effect sizes were reported using Cohen’s d (41) for parametric tests and rank-biserial correlation for non-parametric tests, along with corresponding 95% confidence intervals.

For classification analysis, a Support Vector Machine (SVM) (42) with a radial basis function (RBF) kernel was implemented using the scikit-learn library (v1.0.2). Feature selection was performed via recursive feature elimination with cross-validation (RFECV) (27) to identify the most predictive biomarkers from the multimodal dataset. Hyperparameters (C = 100, γ = 0.1) were optimized through five-fold cross-validation. Class imbalance was addressed by applying weighted training strategies. Model performance was assessed based on accuracy, the area under the receiver operating characteristic curve (AUC-ROC), and permutation testing with 1,000 iterations to establish statistical significance of the classification outcomes.

## 3 Results

### 3.1 Demographic data

As shown in **Table 2**, the study included 115 participants in total, comprising 64 healthy controls (HCs) and 51 adolescents with MDD. Gender distribution was similar across groups, with 35.29% males in the MDD group and 45.31% in the HC group (*p* = 0.278). As expected, depression severity, measured by the CES-D scale, was markedly higher in the MDD group (24.82 ± 6.16) compared to HCs (9.53 ± 4.39; p < 0.001), which confirmed the clinical validity of the group classification.

**Table 2:** Sample characteristics.

Characteristic	HCs (n=64)	MDD (n=51)	Overall (n=115)	*p-value*
Age, mean(SD)	14.14(2.22)	16.27(1.80)	15.09(2.30)	*p*<0.001
Gender, n(%)				*p*=0.278
Male	29(45.31%)	18(35.29%)	47(40.87%)	
Female	35(54.69%)	33(64.71%)	68(59.13%)	
Depression symptoms				*p*<0.001
CES-D	9.53(4.39)	24.82(6.16)	16.31(9.25)	

### 3.2 Multivariate analysis

In the domain of EEG measurements, the MDD group exhibited a significantly elevated theta/beta ratio compared to the control group (MDD: 2.52 [1.53, 3.35]; Control: 1.91 [1.31, 2.47]; *p* = .001) (**Figure 1a**). Regarding eye-tracking metrics, the depression group showed a notably reduced saccade count (MDD: 216 [171, 256]; Control: 245 [184.25, 287.25]; *p* = .046) (**Figure 1b**). Additionally, the mean fixation duration was significantly longer in the MDD group (0.76 [0.67, 0.85] s) relative to controls (0.68 [0.54, 0.83] s; *p* = .046) (**Figure 1c**). The average saccade distance was also greater in the depression group (325.59 ± 73.70 px) than in controls (288.82 ± 57.25 px), yielding a t-value of 9.066 (*p* = .003) (**Figure 1d**). Likewise, the mean saccade duration was significantly increased in the MDD group (0.21 [0.20, 0.23] s) compared to the control group (0.20 [0.17, 0.22] s; *p* = .004) (**Figure 1e**). In terms of autonomic nervous system activity, participants with MDD demonstrated a significantly elevated low-frequency to high-frequency (LF/HF) ratio (MDD: 0.39 [0.31, 0.60]; Control: 0.34 [0.17, 0.46]; *p* = .003) (**Figure 1f**), indicating altered heart rate variability patterns.

### 3.3 Correlation analysis

To mitigate the risk of Type I errors arising from multiple comparisons between physiological indicators and depression scores, a Bonferroni correction (43) was applied. The adjusted significance threshold was calculated as: α_corrected = α_original/m, where m represents the product of the number of EEG indicators, eye-tracking, HRV features, and the number of depression scales used.

**Figure 2:**
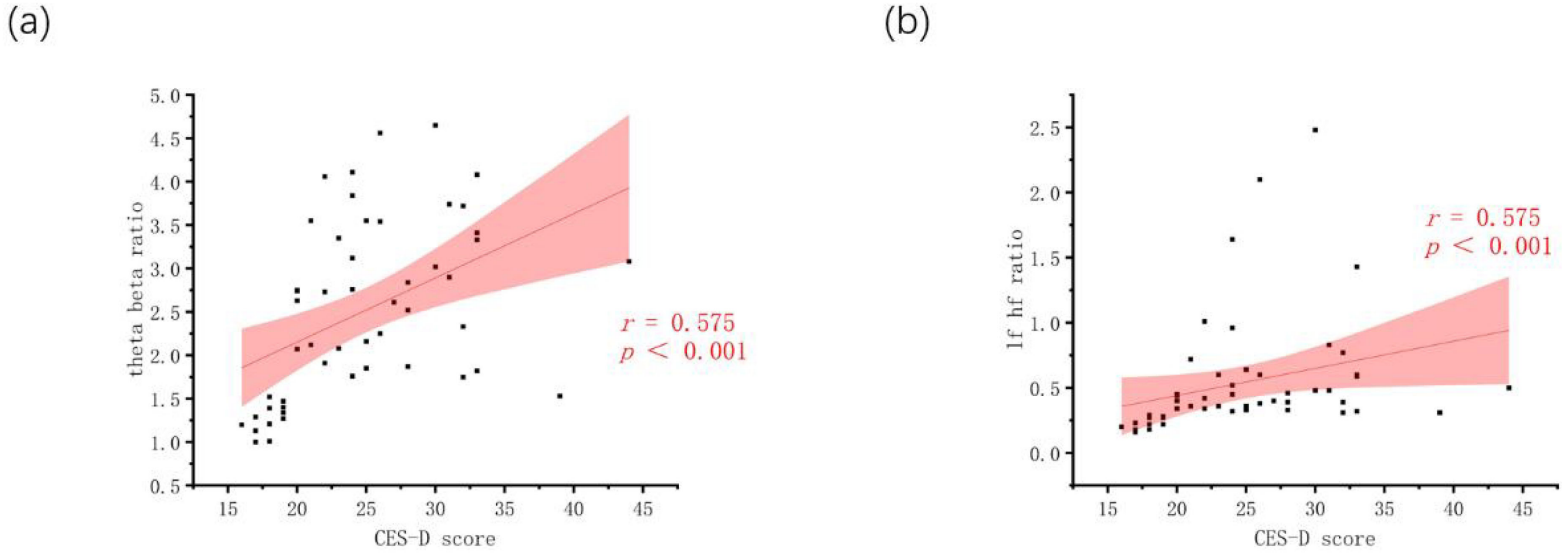
Correlation plots. **(a)** Theta beta ratio vs. CES-D; **(b)** LF/HF ratio vs. CES-D.

In this study, depression severity was evaluated using the CES-D scale (35). Based on group-level comparisons, one EEG measure, four eye-tracking metrics, and one HRV variable were identified as significantly different between groups, yielding an adjusted alpha level of α_corrected = 0.0125. Within the MDD group, CES-D scores were positively correlated with the theta/beta ratio (r = 0.575, *p* <.001) (**Figure 2a**) and the LF/HF ratio (r = 0.575, *p* <.001) (**Figure 2b**), both exceeding the corrected significance threshold. In contrast, no significant correlations were observed between CES-D scores and average saccade distance (*p* = .180), saccade duration (*p* = .287), saccade count (*p* = .443), or fixation duration (*p* = .243).

### 3.4 Classification performance

**Figure 3:**
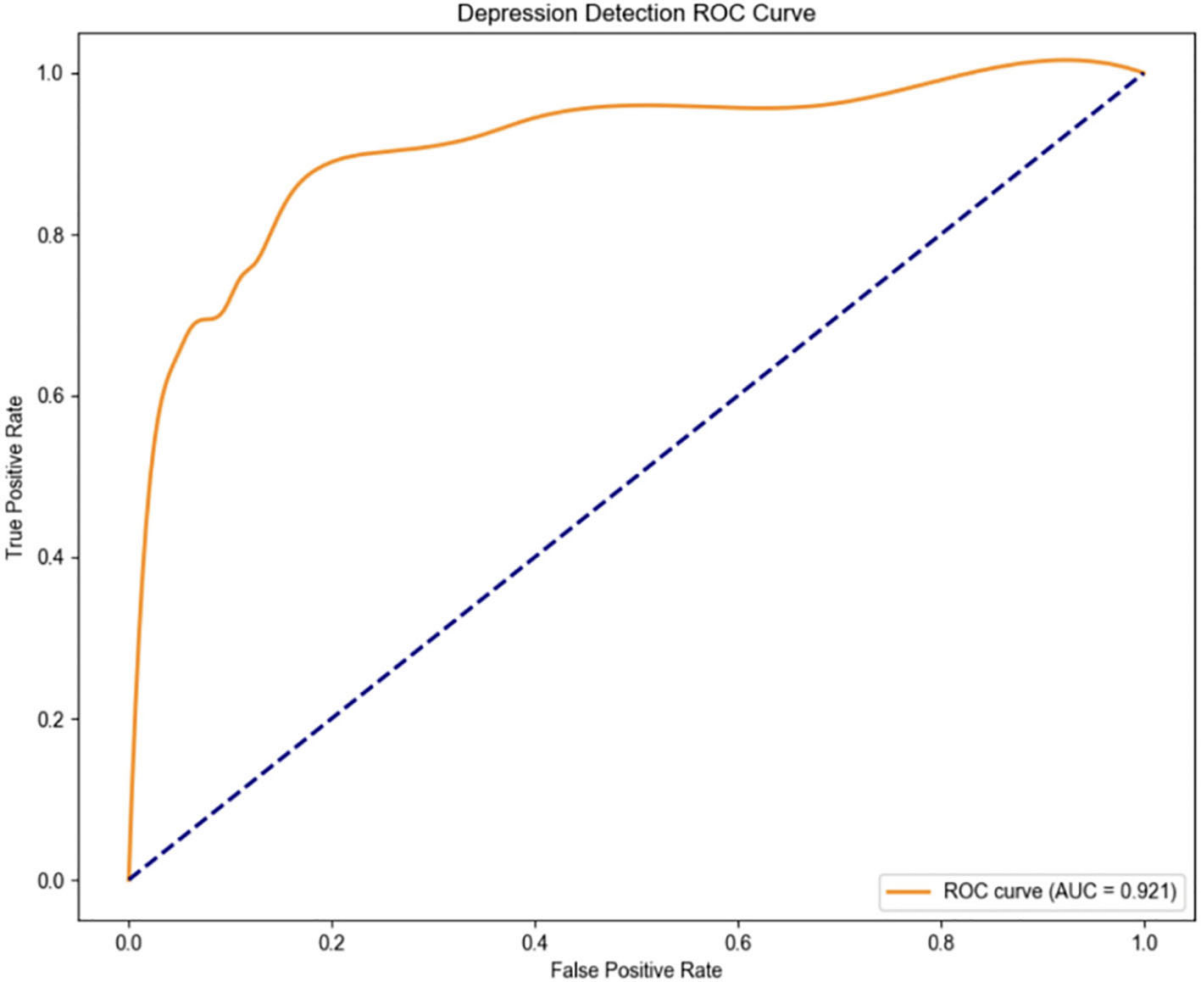
ROC curve (“normal” class weight = 1, “depression” class weight = 1).

An SVM-based binary classification model (**Figure 3**) was developed to differentiate individuals with MDD from healthy controls. The model was optimized via five-fold cross-validation, using a radial basis function (RBF) kernel with hyperparameters set to C = 100 and γ = 0.1. Only physiological features that demonstrated statistically significant differences between groups (*p* <.01) were included as input features. Under the equal class weight condition, **Figure 3** illustrates that the model achieved an overall accuracy of 81.74% and an area under the ROC curve (AUC) of 0.921, indicating strong classification performance.

**Figure 4:**
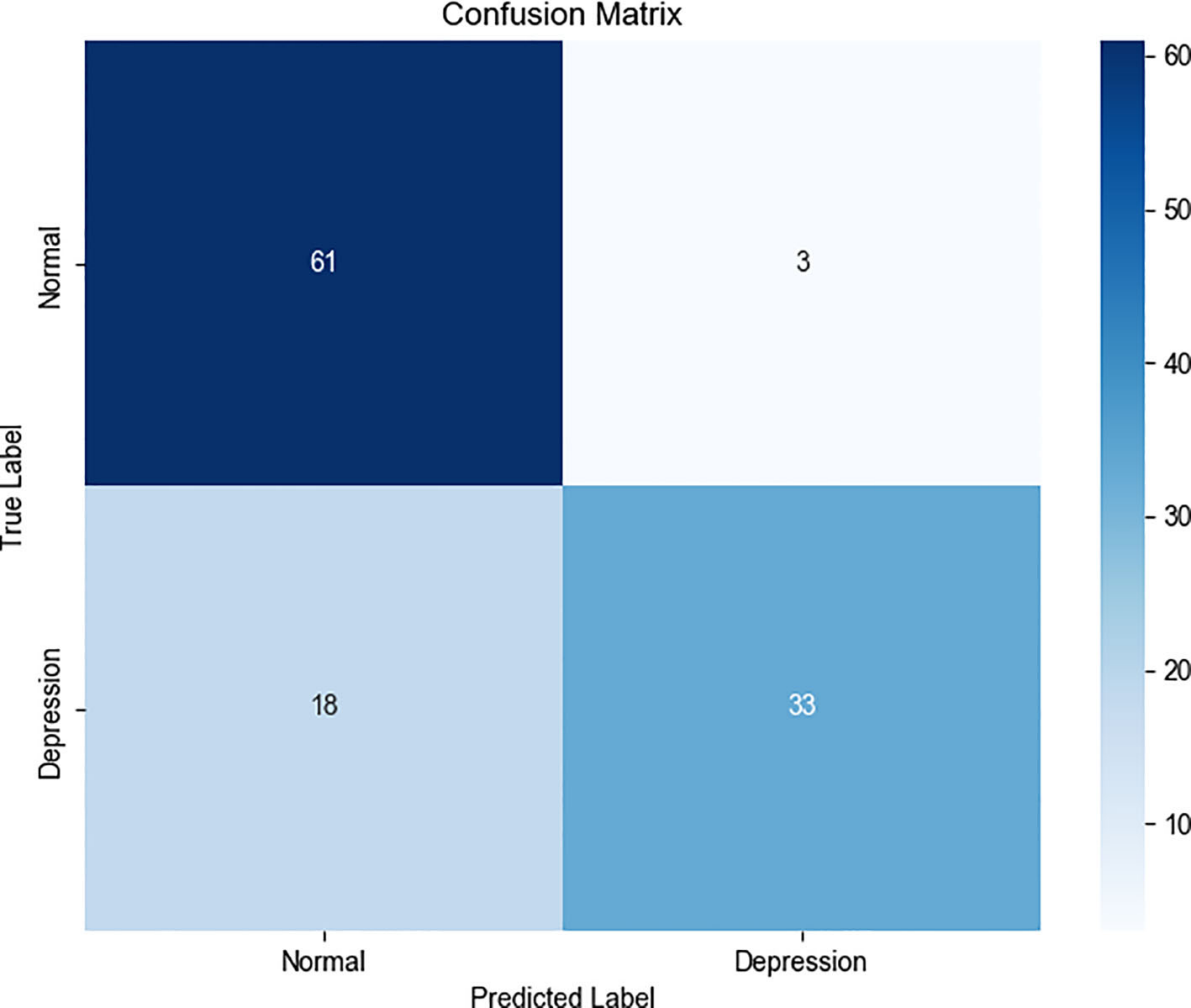
Confusion matrix (“normal” class weight = 1, “depression” class weight = 1).

**Figure 5:**
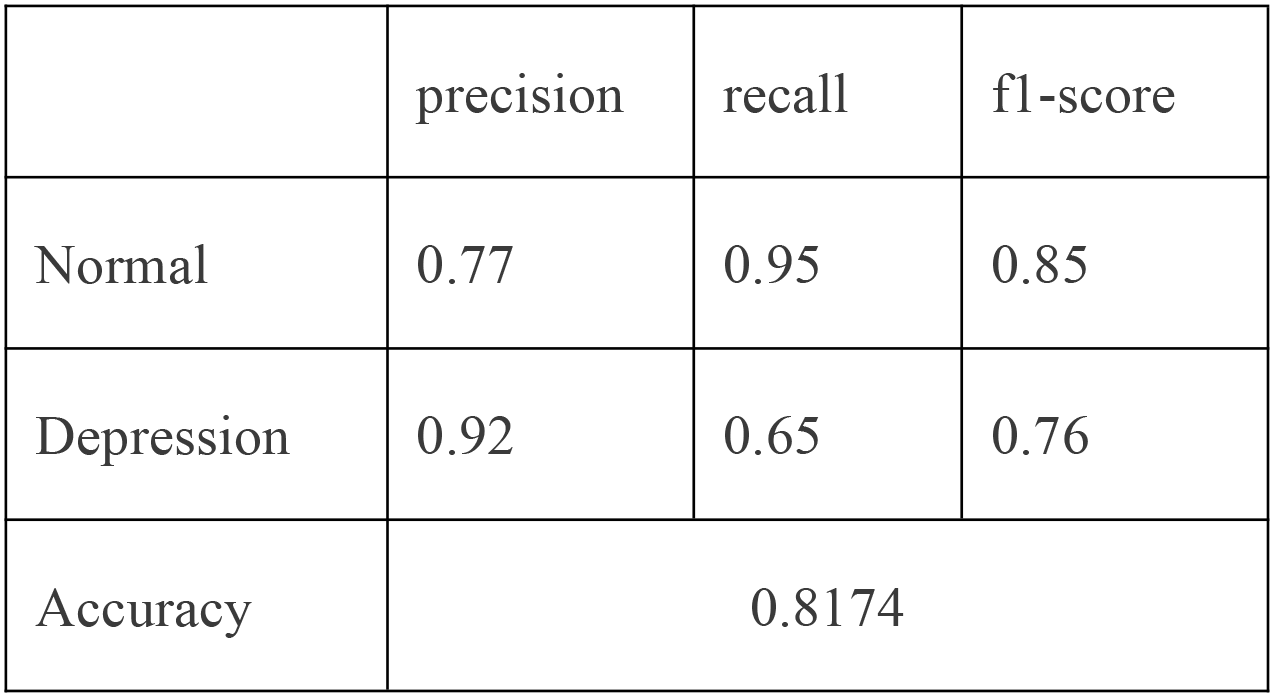
Classification report (“normal” class weight = 1, “depression” class weight = 1).

The classification report and confusion matrix (**Figure 4**) revealed that the model performed well in identifying the control group, while the recall rate for the depression group was relatively lower (Recall = 0.65) (**Figure 5**), suggesting a degree of under-diagnosis.

**Figure 6:**
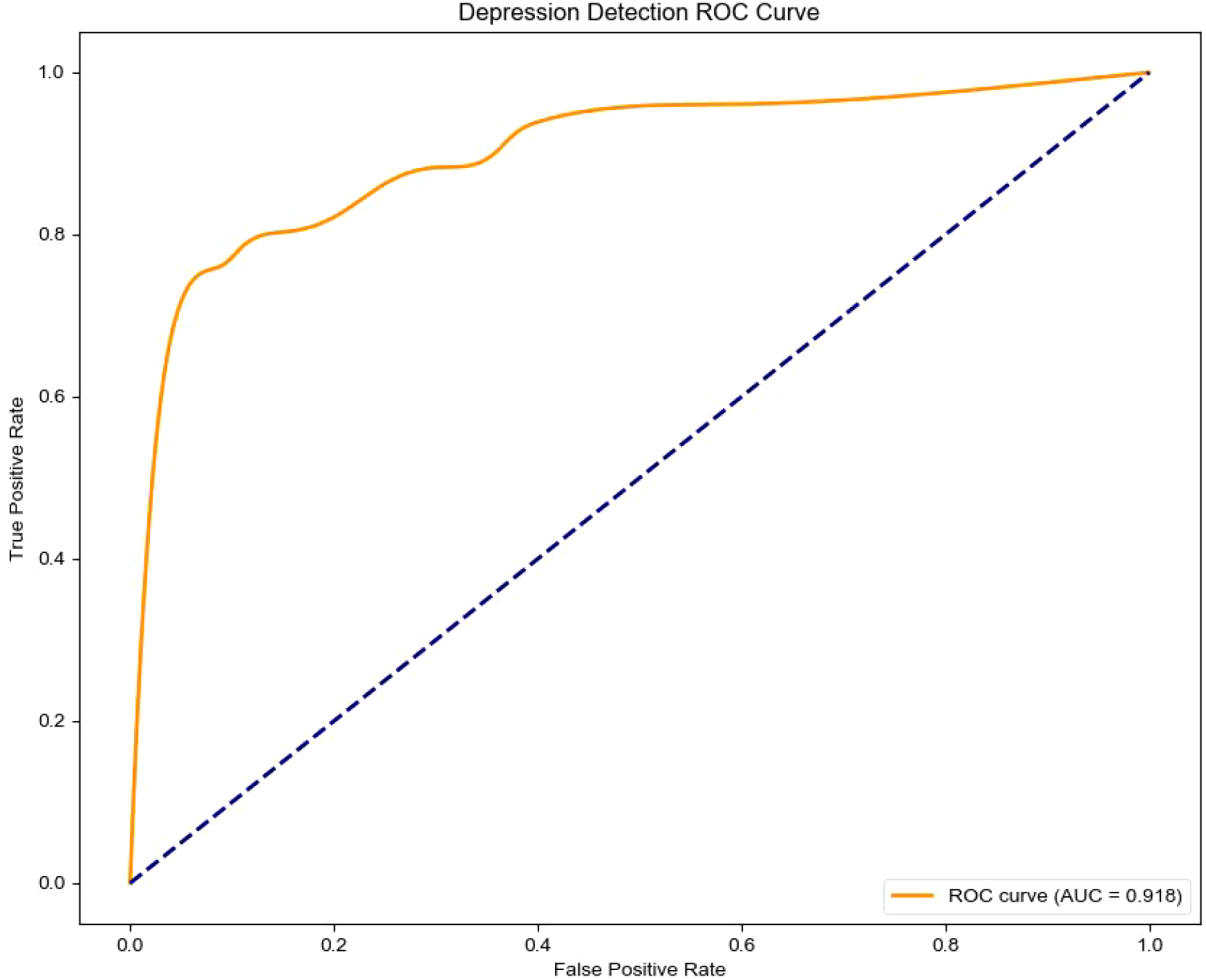
Confusion matrix (“normal” class weight = 1, “depression” class weight = 1.25).

**Figure 7:**
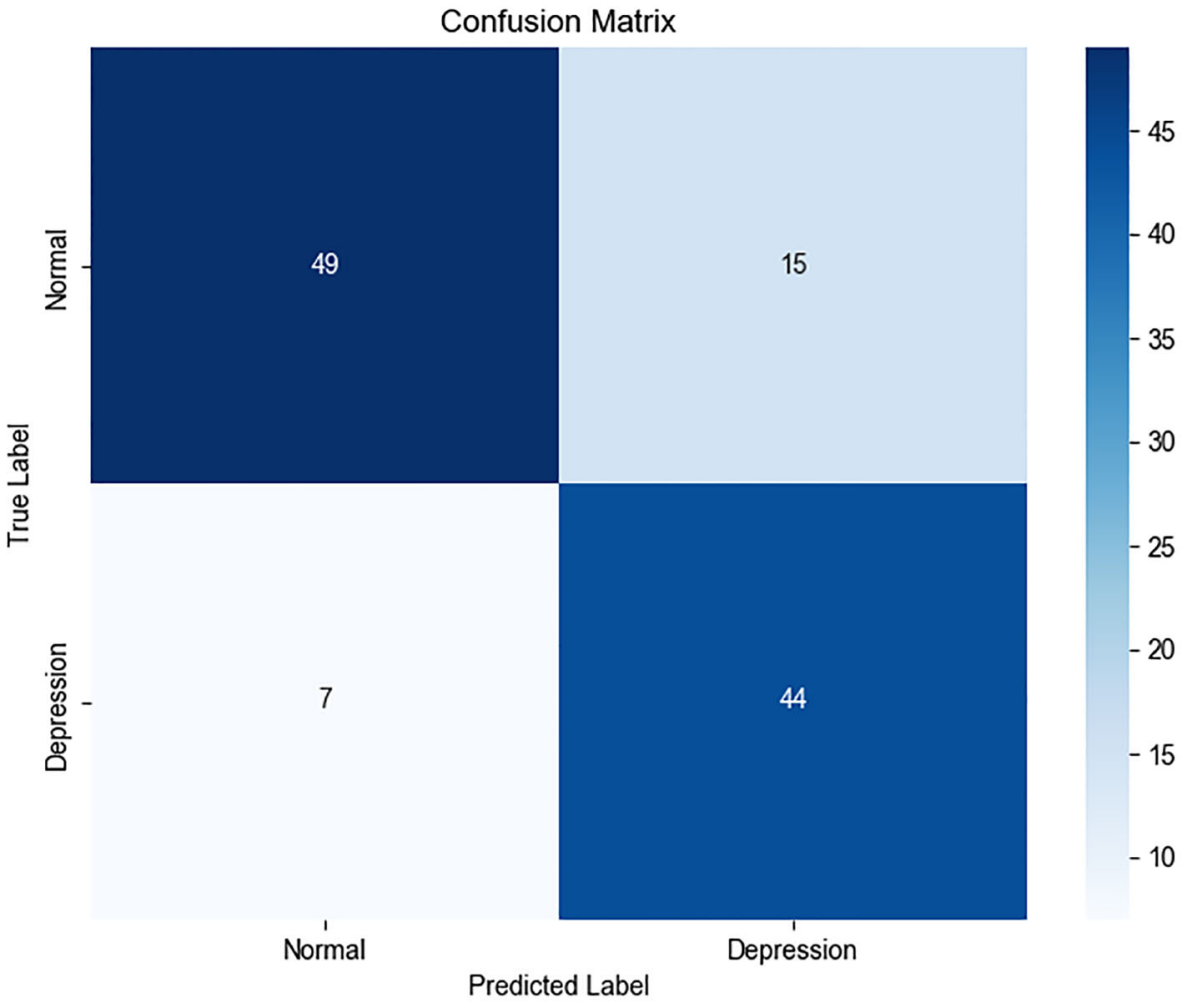
Confusion matrix (“normal” class weight = 1, “depression” class weight = 1.25).

**Figure 8:**
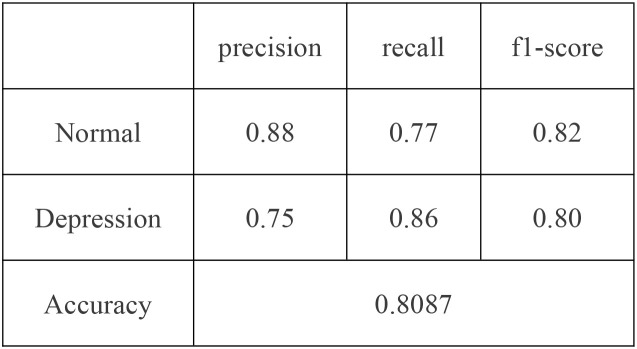
Classification report (“normal” class weight = 1, “depression” class weight = 1.25).

In practical scenarios, such as in healthcare where missing a positive case is more critical than making a false positive, we can increase the weight of the “depression” class—for example, raising its weight to 1.25 as displayed in **Figure 6** - ROC curve and **Figure 7** - Confusion matrix. This adjustment can improve the recall and other relevant metrics, as shown **Figure 8**:

In summary, the proposed model demonstrates robust performance in identifying individuals with depression. For practical deployment, the classification strategy can be flexibly adjusted by tuning class weights according to application-specific risk preferences.

## 4 Discussion

### 4.1 Key findings

This study identified robust physiological markers of adolescent MDD through a novel, VR-integrated multimodal assessment framework. Compared to healthy controls, adolescents with MDD exhibited elevated theta/beta EEG ratios, altered ocular motility patterns—characterized by reduced saccade counts but increased fixation duration and saccade distance/duration—and heightened LF/HF ratios in HRV. Among these, four features—theta/beta ratio, LF/HF ratio, saccade distance, and saccade duration—demonstrated significant correlations with depression severity (p <.0125, Bonferroni-corrected). When integrated into a SVM model, these physiological indicators yielded an overall classification accuracy of 81.7% (AUC = 0.921), underscoring their potential as objective biomarkers for MDD. These findings are consistent with established neurophysiological models of depression implicating impaired cortical arousal regulation and autonomic dysfunction. Importantly, this work advances previous studies by incorporating multimodal signals within an ecologically valid, immersive environment (44).

### 4.2 Comparison with prior works

Recent advances in machine learning have led to substantial progress in the objective identification of major depressive disorder (MDD), particularly through the use of multimodal physiological data such as EEG, eye-tracking, and autonomic indicators. Multiple studies have shown that classification models achieving over 80% accuracy are not only feasible but also meet current expectations for psychiatric screening tools. For instance, a systematic review and meta-analysis of resting-state functional MRI-based machine learning models reported classification accuracies ranging from approximately 75% to 89%, depending on data modality and model configuration, supporting the reliability of accuracies within this range for MDD diagnosis (45). In addition, research leveraging eye-tracking features within virtual reality environments has demonstrated high diagnostic precision; one study reported an accuracy of 86% and an F1 score of 0.92 in distinguishing individuals with depression from controls using eye movement features alone (46). Furthermore, the inclusion of eye-tracking biomarkers has been shown to significantly improve the diagnostic performance of models identifying depression with mixed symptom presentations (47). Beyond behavioral signals, multimodal physiological studies integrating EEG, eye-tracking, and galvanic skin response (GSR) have consistently demonstrated robust performance, with classification accuracies close to 80% (48). A broader synthesis of machine learning applications in psychiatry also highlights that diagnostic models in this field commonly operate within the 80–90% accuracy range, reaffirming this threshold as a credible benchmark (49).

As a consequence, these collective findings reinforce the validity of the current study’s classification results. Our model’s accuracy of 81.7% aligns well with prior research and demonstrates that a performance level above 80% is both empirically grounded and clinically acceptable for MDD identification. Moreover, our results highlight the added screening value of combining heterogeneous physiological markers for adolescent depression detection, extending beyond the limitations of unimodal approaches. While prior machine learning models have reported classification accuracies as high as 88.6% (28), these typically relied on static, resting-state data with limited real-world applicability. By contrast, our VR-based protocol captures physiological reactivity during interactive, emotionally salient tasks, thereby enhancing ecological validity and reflecting dynamic, task-evoked psychopathology. Moreover, the performance of our classification model aligns with recent trends in the use of VR-based diagnostics for other psychiatric conditions, such as schizophrenia (50), suggesting broader applicability of immersive digital tools for mental health evaluation. The integration of VR and physiological monitoring thus represents a meaningful evolution in adolescent depression screening.

### 4.3 Implications

The results of this study underscore the potential utility of VR-enhanced multimodal assessment as a novel strategy for early identification of adolescent MDD. First, the model’s high classification accuracy (81.7%) supports its feasibility for implementation in school-based screenings or primary care settings, particularly for populations reluctant to disclose symptoms through self-report. Second, the reliance on objective physiological indicators enhances diagnostic reliability and may prove useful for longitudinal monitoring during treatment. Third, the approach may help disambiguate MDD from clinically overlapping disorders such as ADHD by leveraging distinct neurophysiological signatures. Finally, the use of VR represents a significant technological advancement, providing a controlled yet immersive environment that encourages naturalistic behavioral expression while maintaining experimental rigor. This balance between ecological validity and standardization positions the method as a promising adjunct to traditional diagnostic protocols.

### 4.4 Limitations and future directions

Despite its promising results, this study has several limitations. First, the sample was demographically limited to Chinese adolescents in a specific region. Even though MDD demonstrates high cross-cultural consistency in both core symptoms and DSM-5 diagnostic criteria (25, 30), future studies should include more diverse populations to evaluate cross-cultural validity and increase applicability. Second, the modest sample size (n=115) may increase the risk of overfitting. To further validate the model, it is necessary to obtain independent datasets from various sites and cohorts to confirm robustness across populations and recording conditions. Third, although the SVM model demonstrated strong performance, interpretability remains a concern. Like many machine learning-based methods, the current approach functions as a “black box,” which may hinder clinical acceptance (51). In the future, scientists or researchers can incorporate explainable AI methods such as SHAP or LIME to enhance transparency and improve clinical adoption. In addition, expanding the feature set to include task-based behavioral or cognitive metrics may further improve predictive performance and offer richer insights into depressive symptomatology.

## 5 Conclusion

This study developed a VR-based multimodal framework combining EEG, HRV, and eye-tracking to identify objective biomarkers of adolescent depression. Key features—such as elevated theta/beta and LF/HF ratios—enabled an SVM classifier to achieve 81.7% accuracy (AUC = 0.921), demonstrating strong diagnostic potential. The integration of immersive environments and physiological signals offers a promising, scalable approach for early depression screening, which also has the potential to be implemented in schools or primary healthcare settings. Future work should improve model interpretability and validate the framework across diverse populations.

## Data Availability

The raw data supporting the conclusions of this article will be made available by the authors, without undue reservation.
